# Visual diagnosis of female genital schistosomiasis in Zambian women from hand-held colposcopy: agreement of expert image review and association with clinical symptoms

**DOI:** 10.12688/wellcomeopenres.18737.2

**Published:** 2023-05-04

**Authors:** Amy Sturt, Henrietta Bristowe, Emily Webb, Isaiah Hansingo, Comfort Phiri, Maina Mudenda, Joyce Mapani, Tobias Mweene, Bruno Levecke, Piet Cools, Govert van Dam, Paul Corstjens, Helen Ayles, Richard Hayes, Suzanna Francis, Lisette van Lieshout, Bellington Vwalika, Eyrun Kjetland, Amaya Bustinduy

**Affiliations:** 1 Department of Obstetrics and Gynecology, University of Lusaka, Lusaka, Zambia; 2 Department of Infectious Diseases, Oslo University Hospital, Oslo, 0450, Norway; 3 University of KwaZulu-Natal, Durban, 4041, South Africa; 4 Department of Medicine, Division of Infectious Diseases, Veterans Affairs Healthcare System, Palo Alto, CA, 94304, USA; 5 Department of Medicine, Division of Infectious Diseases and Geographic Medicine, Stanford University, Palo Alto, California, 94305, USA; 6 Kings College Hopsital NHS Foundation Trust, London, SE5 9RS, UK; 7 MRC International Statistic and Epidemiology Group, London School of Hygiene & Tropical Medicine, London, WC1E 7HT, UK; 8 Department of Obstetrics and Gynecology, Livingstone Central Hospital, Livingstone, Zambia; 9 Zambart, Lusaka, Zambia; 10 Department of Translational Physiology, Infectiology, and Public Health, Ghent University, Merelbeke, B-9820, Belgium; 11 Department of Diagnostic Sciences, Faculty of Medicine and Health Sciences, Ghent University, Ghent, B-9000, Belgium; 12 Department of Parasitology, Leiden University Medical Center, Leiden, 2300 RC, The Netherlands; 13 Department of Cell and Chemical Biology, Leiden University Medical Center, Leiden, 2300 RC, The Netherlands; 14 Department of Clinical Research, London School of Hygiene & Tropical Medicine, London, WC1E 7HT, UK

**Keywords:** Female genital schistosomiasis, Schistosoma haematobium, hand-held colposcopy

## Abstract

**Background: **Female genital schistosomiasis (FGS) can occur in *S. haematobium* infection and is caused by egg deposition in the genital tract. Confirming a diagnosis of FGS is challenging due to the lack of a diagnostic reference standard. A 2010 expert-led consensus meeting proposed visual inspection of the cervicovaginal mucosa as an adequate reference standard for FGS diagnosis. The agreement of expert human reviewers for visual-FGS has not been previously described.

**Methods:** In two Zambian communities, non-menstruating, non-pregnant, sexually-active women aged 18-31 years participating in the HPTN 071 (PopART) Population-Cohort were enrolled in a cross-sectional study. Self-collected genital swabs and a urine specimen were collected at a home visit; trained midwives performed cervicovaginal lavage (CVL) and hand-held colposcopy at a clinic visit. *S. haematobium* eggs and circulating anodic antigen (CAA) were detected from urine. Two senior physicians served as expert reviewers and independently diagnosed visual-FGS as the presence of sandy patches, rubbery papules or abnormal blood vessels in cervicovaginal images obtained by hand-held colposcopy. PCR-FGS was defined as *Schistosoma *DNA detected by real-time PCR in any genital specimen (CVL or genital swab).

**Results:** Of 527 women with cervicovaginal colposcopic images, 468/527 (88.8%) were deemed interpretable by Reviewer 1 and 417/527 (79.1%) by Reviewer 2. Visual-FGS was detected in 35.3% (165/468) of participants by expert review of colposcopic images by Reviewer 1 and in 63.6% (265/417) by Reviewer 2. Cohen’s kappa statistic for agreement between the two reviewers was 0.16, corresponding to "slight" agreement. The reviewers made concordant diagnoses in 38.7% (204/527) participants (100 negative, 104 positive) and discordant diagnoses in 31.8% (168/527) participants.

**Conclusions:** The unexpectedly low level of correlation between expert reviewers highlights the imperfect nature of visual diagnosis for FGS based on cervicovaginal images. This finding is a call to action for improved point-of-care diagnostics for female genital schistosomiasis.

## Introduction

Female genital schistosomiasis (FGS), primarily caused by *S. haematobium* infection, is a neglected tropical disease associated with poverty, inadequate sanitation, and limited access to safe drinking water ^1, 2^. FGS occurs when schistosome eggs destined for excretion via the urinary bladder are deposited in the female genital tract. These tissue-deposited eggs can be associated with characteristic genital mucosal lesions and can present with genitopelvic findings including contact bleeding ^3^, abnormal vaginal discharge ^4^, and in some cases, infertility ^5^. Visual-FGS refers to the identification of these characteristic mucosal changes, such as sandy patches (grainy and homogeneous), rubbery papules, and abnormal blood vessels by visual inspection of the cervicovaginal mucosa ^3^. The visual detection of FGS-associated lesions requires the insertion of a vaginal speculum, a good light source, and a lens providing adequate magnification ^6^. A standard colposcope has traditionally been used in research settings for visual-FGS diagnosis, but the bilharzia and HIV (BILHIV) study demonstrated recently that hand-held colposcopy could also be used to decentralize colposcopy services ^6– 9^.

Confirming a diagnosis of FGS is challenging as there is not a widely accepted diagnostic reference standard for research, diagnosis, and screening ^2^. A 2010 expert-led consensus meeting proposed visual inspection of the cervicovaginal mucosa as an adequate reference standard for FGS diagnosis ^10^. However, the mucosal changes in visual-FGS are non-specific and have also been associated with herpes simplex virus, human papillomavirus (HPV) infection, and cervical precancer ^3^. Diagnostic methods that are not adequately specific for FGS diagnosis may lead to over-treatment with praziquantel and may overlook the diagnosis and treatment of sexually transmitted infection (STI) and cervical cancer. Although there is little evidence of praziquantel resistance in humans ^11^, indiscriminate treatment may theoretically increase the risk of the development of praziquantel resistance ^12^. Since cervicovaginal visualization is widely promoted ^13^ for FGS screening and diagnosis, we aimed to use BILHIV study data to further evaluate the agreement of human expert reviewers for the diagnosis of visual-FGS. Secondary aims were to evaluate the association between visual-FGS and abdominal, genitourinary, and reproductive manifestations as well as evaluating *Schistosoma* diagnostic methods for their association with the presence of visual-FGS.

## Methods

### Study setting and participants

The cross-sectional bilharzia and HIV (BILHIV) study ^9^ was nested in the HPTN 071 (PopART) cluster randomized trial in Zambia ^14^. The *S. haematobium* is endemic in Zambia, and while more data are needed, prevalences ranging between 14 – 76% were reported in a recent systematic review ^15^. The HPTN 071 (PopART) trial was a cluster randomized trial assessing the impact of an HIV-1 combination prevention package including “universal test and treat” ^14^. As previously described, after the 36-month HPTN 071 (PopART) visit, community workers made home visits to women expressing interest in the BILHIV study ^9^. Between January and August 2018, eligible women who were 18–31 years old, not pregnant, sexually active, and resident in one of two urban communities that participated in HPTN 071 (PopART) in Livingstone, Zambia were enrolled in the BILHIV study. The primary aim of the BILHIV study was to compare the performance of genital self-sampling (cervical and vaginal swabs) to clinic-based cervicovaginal lavage (CVL) for the detection of *Schistosoma* DNA by quantitative PCR (qPCR) as previously described ^9^. A specific pre-specified BILHIV study objective (the subject of the current manuscript) was to compare agreement of expert review of images obtained through hand-held colposcopy for the diagnosis of visual-FGS.

### Home and clinic-based sample collection

As previously described, the home visit included written informed consent, a questionnaire, genital self-sampling (cervical and vaginal), and collection of a urine specimen ^9^. There were no restrictions on the timing of urine self-sample collection, and 69.5% (419/603) of the total BILHIV study samples were performed between 9:00 and 14:00 ^9^. Enrolled women who were not currently menstruating were then invited to attend Livingstone Central Hospital cervical cancer clinic, where midwives collected CVL. After speculum insertion, normal saline (10 mL) was flushed across the cervix and vaginal walls for one minute with a bulb syringe and CVL fluid was collected from the posterior fornices.

### Hand-held colposcopy and image review

At the clinic, cervicovaginal images were captured with a portable colposcope (EVA System, MobileODT, Tel Aviv, Israel) according to a standardized protocol. Per the protocol, trained midwives evaluated the cervix, anterior fornix, posterior fornix, left and right lateral cervix and vaginal walls and captured images of each location using the zoom and lighting functions in the Mobile ODT colposcope. Two senior physicians who have training and expertise in colposcopy and FGS served as expert reviewers. Digital images were independently evaluated by the expert reviewers for any of the four recognized FGS cervicovaginal manifestations: grainy sandy patches, homogenous yellow sandy patches, rubbery papules, and abnormal blood vessels ^16^. At their discretion, expert reviewers could exclude images that they felt could not be evaluated due to technical issues, image quality, or limited cervical visualization. If any of the four recognized FGS cervicovaginal manifestations was present, the participant was categorized as “visual-FGS”. If none of the four cervicovaginal manifestations were present the participant was categorized as “visual-FGS not detected” ^16^. The expert reviewers were both senior practicing physicians at the Professor level, who have training and expertise in standard colposcopy. Reviewer 1 (EFK) is full-time FGS researcher and an infectious diseases physician and Reviewer 2 (BV) is an obstetrician and gynecologist who regularly analyses images for cervical cancer. Both reviewers have extensive practical and research-based expertise in evaluating and diagnosing FGS in endemic settings. Additionally, both reviewers contributed as authors of the 2015 WHO FGS Pocket Atlas ^16^. Each reviewer was informed of the study setting and methods, but both were blinded to the study participants’ FGS and *Schistosoma* status.

Women with at least one of the visual manifestations of FGS ^3, 16^ or with any positive urine or genital *Schistosoma* diagnostic result were treated free-of-charge with 40 mg/kg praziquantel. Testing for STIs was not performed at the point-of-care and participants with suspected STIs were offered syndromic management, as per local guidelines ^17^. In line with national and local clinic protocols adapted to real-world resource limitations, human papillomavirus (HPV) testing was not performed.

In parallel with BILHIV study procedures, participants could choose to engage in free cervical cancer screening using the visual inspection with acetic acid (VIA) technique. In the subset of women who engaged in cervical cancer screening, midwives applied 3-5% acetic acid to the cervix after CVL collection, as previously described ^18^. An opaque white reaction was classified as positive and no change as negative ^19^. Images for FGS analyses were taken before application of acetic acid. Images for cervical cancer screeing were taken after application of acetic acid.

### Urine microscopy, and circulating anodic antigen

Up to 60mL of fresh urine was centrifuged and examined by microscopy for *S. haematobium* eggs. The participant was considered to have urinary schistosomiasis if a pellet contained at least one *S. haematobium* egg ^9^. All study specimens were stored at -80°C. A lateral flow assay utilizing up-converting reporter particles for the quantification of circulating anodic antigen (CAA) was performed on urine samples, as previously described ^9, 20^. Analyzing the equivalent of 417 μL urine (wet reagent, UCAA ***hT*** 417), a test result indicating a CAA value >0.6 pg/mL was considered positive ^21^.

### qPCR for detection of Schistosoma DNA

DNA extraction, amplification and detection of the *Schistosoma*-specific internal-transcribed-spacer-2 (ITS-2) target by real-time (qPCR) was performed at Leiden University Medical Center, as previously described, using 200 µL of CVL, cervical or vaginal swab fluid ^9, 22^.

### Other infections

Due to budgetary constraints, a subset of participants was evaluated for STI. As previously described, all participants with FGS and all participants with probable FGS were selected for characterization of the cervicovaginal microbiota and STI by qPCR on cervical swabs ^23^. Three FGS-negative participants were selected for every FGS and probable FGS participant using a random number generator. The FGS-negative participants were frequency-matched by age to participants with FGS ^23^. Laboratory-based fourth-generation HIV-1 testing (Abbott Architect HIV Ag/Ab Combo Assay) was performed for HPTN 071 (PopART) Population Cohort participants at each study visit ^14^. STIs were quantified among a subset of participants by qPCR using the S-DiaCTNG ^TM^ (for *C. trachomatis* and *N. gonorrhea*) and S-DiaMGTV ^TM^ kits (for *M. genitalium* and *T. vaginalis*) (Diagenode Diagnostics, Seraing, Belgium) on DNA from cervical swabs at Ghent University (Ghent, Belgium) according to the manufacturer’s instructions.

### Consent

The study was approved by the University of Zambia Biomedical Research Ethics Committee (011-08-17), the Zambia National Health Research Authority and the London School of Hygiene and Tropical Medicine Ethics Committee (14506). Permission to conduct the study was given by Livingstone District Health Office and the Livingstone Central Hospital superintendent.

### Statistical methods

The planned sample size of the BILHIV study was based on calculations related to the primary BILHIV study objective, as previously described ^9^. Participant characteristics were summarized by median and interquartile range (IQR) for continuous variables, and by frequency and percentage for categorical variables. Participants missing data for a specific variable were excluded from analysis involving that variable. The primary analysis evaluated the agreement between the two expert reviewers using Cohen’s kappa statistic. A secondary analysis evaluated the association between visual-FGS (exposure) and abdominal, genitourinary, and reproductive manifestations (outcomes). Crude associations were evaluated using chi-squared tests, and logistic regression was used to calculate crude and adjusted odds ratios (OR) for the association of visual-FGS with clinical manifestations; this was done separately for each expert reviewer’s diagnosis of visual-FGS. In this study we employed various diagnostic tests to evaluate urinary *Schistosoma* infection (CAA and urine microscopy), and FGS (portable colposcopy, and *Schistosoma* DNA on CVL and genital swabs) as previously described ^23– 25^. Another secondary analysis evaluated each diagnostic method for its association with the presence of visual-FGS, separately for each expert reviewer. Due to small numbers, for evaluating the association of visual-FGS with PCR-FGS, we used a composite definition of PCR-FGS or “any positive genital PCR”, defined as any positive cervical or vaginal swab or CVL specimen. Chi-squared tests were used to assess crude associations, and logistic regression was used to calculate crude and age-adjusted odds ratio (OR) of the various *Schistosoma* and FGS diagnostics with the presence or absence of visual-FGS. We were unable to adjust for other potential confounders due to small numbers, particularly for STI and cervical pre-cancer status which were collected on a sub-set of participants. For both secondary analyses, exact logistic regression was used for analyses where 5 or fewer participants in a particular exposure category had the outcome. Due to the exploratory nature of these analyses, we did not adjust for multiple comparisons. Data were analyzed using STATA 15.1 (Stata Corporation, College Station, TX).

## Results

### Baseline characteristics and demographics

**Figure 1.**
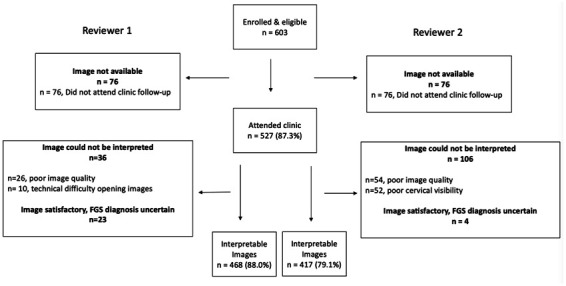
Flowsheet of cervicovaginal image review after hand-held colposcopy in BILHIV study participants.

The BILHIV study enrolled 603 eligible women, 527 (87.4%) of whom had cervicovaginal images captured by portable colposcopy. Of the 527 women with images, 468 (88.8%) were deemed interpretable by Reviewer 1 and 418 (79.3%) by Reviewer 2 ( Figure 1). Each reviewer designated a proportion of images uninterpretable, leading to differences in denominators. The median age of the participants was 24 years (range 22 – 28) and 323 (61.3%) had attended some secondary school ( Table 1). The majority of participants were married, had previously been pregnant, and had been sexually active within the last six months. There was no association between visual FGS, as identified by any expert review, and current or childhood water contact.

**Table 1.** Demographics and reproductive health characteristics of the BILHIV study participants who underwent portable colposcopy (n=527).

Participant Characteristics		Study population (n=527)
Age in years – Median (IQR)		24 (22 – 28)
Marital status	Single	213 (40.4)
	Married or cohabitating	292 (55.4)
	Divorced or separated	22 (4.2)
Education (highest level)	None or any primary school	155 (29.4)
	Any secondary school	323 (61.3)
	Trade, degree or higher	49 (9.3)
District	Community A	290 (55.0)
	Community B	237 (45.0)
Household members	1-3	162 (30.7)
	4-5	210 (39.9)
	6+	155 (29.4)
Employment status	Unemployed	363 (68.9)
	Employed	164 (31.1)
Current water contact	None	447 (84.8)
	Any	80 (15.2)
Childhood water contact	None	151 (28.7)
	Any	376 (71.3)
**Reproductive Health Characteristics**		
Age at sexual debut (years) *	8-16	221 (42.0)
	17-19	228 (43.4)
	20-24	77 (14.6)
Lifetime sexual partners	1	145 (27.5)
	2	134 (25.4)
	3	108 (20.5)
	4+	140 (26.6)
Prior pregnancy *	No	74 (14.1)
	Yes	452 (85.9)
Currently sexually active **	No	65 (12.4)
	Yes	460 (87.6)
Condom use with last sex ^†^	No	381 (73.7)
	Yes	136 (26.3)
HIV-1 infection ^††^	Not detected	407 (77.8)
	Detected	116 (22.2)
Any STI ^∼^	Not detected	138 (63.8)
	Detected	73 (34.6)
Any Hormonal Contraception ^∼∼^	No	201 (38.1)
	Yes	326 (61.9)
VIA ^‡^	Negative	213 (89.9)
	Positive	24 (10.1)

*Participants who responded with “no answer” (n=1) are not shown in the table **Defined as any sexual activity in the last 6 months; Participants who responded with “no answer” (n=2) are not shown in the table †Participants who responded with “no answer” (n=10) are not shown in the table ††Participants with missing data (n=4) are not shown in the table ∼STI were evaluated in a sub-set of women from the BILHIV study, missing values (n=316) are not shown in the table) ∼∼Any hormonal contraception is defined as use of injectable agents, implants, or oral contraceptive pills ‡VIA results were not collected in the BILHIV study and were not available for all participants, participants with missing data (n=366) are not shown in the table

### Prevalence of visual-FGS and interrater agreement

Visual-FGS was detected in 35.3% (165/468) of participants by expert review of digital images from hand-held colposcopy by Reviewer 1 and in 63.6% (265/417) by Reviewer 2. The Cohen’s kappa statistic for interrater agreement between the two expert reviewers was 0.16, corresponding to "slight" agreement ( Table 2). The reviewers made concordant diagnoses in 38.7% (204/527) participants (100 concordant negative, 104 concordant positive) and discordant diagnoses in 31.8% (168/527) cases (reviewer 1 positive, reviewer 2 negative in 32; reviewer 2 positive and reviewer 1 negative in 136). Both reviewers agreed 14 images were unevaluable. A further 26.7% (141/527) images were discordant in evaluability by the expert reviewers (Reviewer 1, n=45; Reviewer 2, n=96).

**Table 2.** Agreement of expert reviewers for the presence or absence of visual-FGS.

	Reviewer 1	FGS not detected	Visual FGS	Not evaluable	Total		
Reviewer 2						Cohen’s Kappa (+ SE) **	Interpretation
Visual FGS not detected		100 (19.0)	32 (6.1)	20 (3.8)	152 (28.8) *		
Visual FGS		136 (25.8)	104 (19.7)	25 (4.7)	265 (50.3) *	0.16 (0.04)	None to slight
Not evaluable		67 (12.7)	29 (5.5)	14 (2.7)	110 (20.9)		
Total		303 (57.5) *	165 (31.3) *	59 (11.2)	527 (100.0)		

FGS – female genital schistosomiasis, SE – standard error *total evaluable for Reviewer 1: visual FGS not detected 303/468 (64.7), visual FGS detected 165/468 (35.3); total evaluable for Reviewer 2: visual FGS not detected 152/417 (36.5), visual FGS detected 265/417 (63.5); **Cohen’s kappa is restricted to those participants where both Reviewer 1 and Reviewer 2 provided a diagnosis

### Visual FGS and *Schistosoma* laboratory tests

Of the 527 participants, 6.1% (32/527) had urinary *S. haematobium* infection, as diagnosed by urine microscopy, and 14.9% (78/525) had a detectable urine CAA. There was no association between *S. haematobium* egg-positive urine microscopy or urine CAA and visual-FGS, as defined by Reviewer 1 or Reviewer 2’s assessmen ( Table 3).

**Table 3.** Associations of *Schistosoma* diagnostics with visual-FGS

Participant Characteristics		Visual FGS not detected	Visual FGS detected	Crude OR	P- value *	Adjusted OR **	P- value ^†^
***Schistosoma* diagnostics** ** (Reviewer 1)**		n=303	n=165				
Eggs on urine microscopy	Not detected	287 (94.7)	151 (91.5)	reference	0.3	reference	0.2
	Detected	16 (5.3)	14 (8.5)	1.66 (0.73 – 3.74)		1.68 (0.74 – 3.81)	
Circulating anodic antigen ~	Not detected	261 (86.1)	131 (80.4)	reference	0.1	reference	0.1
	Detected	42 (13.9)	32 (19.6)	1.52 (0.92 – 2.52)		1.49 (0.91 – 2.51)	
Any positive genital PCR	DNA not detected	288 (95.1)	155 (93.9)	reference	0.8	reference	0.7
	DNA detected	15 (4.9)	10 (6.0)	1.24 (0.54 – 2.82)		1.27 (0.55 – 2.94)	
***Schistosoma* Diagnostics** **(Reviewer 2)**		n=152	n=265				
Eggs on urine microscopy ^††^	Not detected	147 (96.7)	243 (91.7)	reference	0.06	reference	0.07
	Detected	5 (3.3)	22 (8.3)	2.65 (0.95 – 9.17)		2.65 (0.95 – 9.19)	
Circulating anodic antigen ~	Not detected	131 (86.7)	215 (81.4)	reference	0.2	reference	0.2
	Detected	20 (13.3)	49 (18.6)	1.49 (0.85 – 2.62)		1.49 (0.85 – 2.63)	
Any positive genital PCR	DNA not detected	146 (96.1)	248 (93.6)	reference	0.3	reference	0.3
	DNA detected	6 (3.9)	17 (6.4)	1.69 (0.64 – 4.33)		1.67 (0.64 – 4.36)	

*Chi squared p-value unless otherwise noted **Adjusted for age †Likelihood ratio test p-value Missing values not included in the table: ~ (n=2) ††Odds Ratios and p-values obtained through exact logistic regression in both crude and adjusted analyses

PCR-FGS, defined as any positive *Schistosoma* qPCR from a genital sample, was diagnosed in 5.0% (30/603) of participants [3.4% (18/527) cervical swab, 2.7% (14/527) vaginal swab, and 2.7% (14/527) CVL]. Further details regarding the operating characteristics of these tests have previously been described ^9^. There was no association between visual-FGS and PCR-FGS, 10 of the 165 women (6.0%) identified by Reviewer 1 as having visual-FGS had PCR-FGS and 17 of the 265 women (6.4%) identified by Reviewer 2 as having visual-FGS had PCR-FGS, compared to 4.9% and 3.9% among women identified by Reviewer 1 and 2 as not having visual-FGS respectively ( Table 3).

### Symptoms

The association between abdominal, genitourinary, and reproductive manifestations and visual-FGS is shown in Table 4. Neither vaginal discharge, vaginal bleeding after sex, the presence of external genital sores, dysuria nor abdominal pain was associated with the presence of visual FGS as diagnosed by either expert reviewer ( Table 4). Self-reported delay in conception was associated with the presence of visual-FGS, as assessed by Reviewer 1, both in crude analysis and after adjusting for age and community of residence (aOR 2.74 [1.29 – 5.83], p<0.01). Visual-FGS, as defined by Reviewer 2’s assessment of hand-held colposcopy images, was associated with hematuria (aOR 4.44 [1.00 – 40.63] p=0.05) and dyspareunia (aOR 1.71 [0.99 – 2.95], p=0.05), albeit with weak evidence of an association ( Table 4).

**Table 4.** Associations of genitourinary and abdominal symptoms with visual-FGS.

Participant Characteristics		Visual FGS not detected	Visual FGS detected	P-value *	Crude OR	P-value	Adjusted OR **	P- value ^†^
**Signs & Symptoms** ** (Reviewer 1)**		n= 303	n=165					
Vaginal discharge	Not present	273 (90.1)	139 (84.2)	0.06	reference	0.06	reference	0.1
	Present	30 (9.9)	26 (15.8)		1.70 (0.97 – 2.99)		1.58 (0.90 – 2.80)	
Dyspareunia	Not present	239 (78.9)	139 (84.2)	0.2	reference	0.2	reference	0.1
	Present	64 (21.1)	26 (15.8)		0.70 (0.42 – 1.15)		0.68 (0.41 – 1.12)	
Vaginal bleeding after sex	Not present	289 (95.4)	153 (92.7)	0.2	reference	0.2	reference	0.3
	Present	14 (4.6)	12 (7.3)		1.62 (0.73 – 3.59)		1.48 (0.66 – 3.31)	
Vaginal sores	Not present	280 (92.4)	154 (93.3)	0.7	reference	0.7	reference	0.6
	Present	23 (7.6)	11 (6.7)		0.87 (0.41 – 1.83)		0.84 (0.40 – 1.77)	
Dysuria	Not present	259 (85.5)	140 (84.9)	0.9	reference	0.9	reference	0.8
	Present	44 (14.5)	25 (15.1)		1.05 (0.62 – 1.79)		0.92 (0.53 – 1.59)	
Hematuria	Not present	295 (97.4)	155 (93.9)	0.07	reference	0.07	reference	0.1
	Present	8 (2.6)	10 (6.1)		2.38 (0.92 – 6.20)		2.18 (0.84 – 5.67)	
Abdominal pain	Not present	217 (71.6)	123 (74.5)	0.5	reference	0.5	reference	0.4
	Present	86 (28.4)	42 (25.5)		0.86 (0.56 – 1.33)		0.82 (0.53 – 1.26)	
Delay in conception ^††^	No	235 (94.4)	111 (86.7)	0.01	reference	0.01	reference	<0.01
	Yes	14 (5.6)	17 (13.3)		2.57 (1.22 – 5.40)		2.74 (1.29 – 5.83)	
**Signs & Symptoms** ** (Reviewer 2)**		n=152	n=265					
Vaginal discharge	Not present	133 (87.5)	236 (89.1)	0.6	reference	0.6	reference	0.6
	Present	19 (12.5)	29 (10.9)		0.86 (0.46 – 1.60)		0.84 (0.45 – 1.57)	
Dyspareunia	Not present	131 (86.2)	208 (78.5)	0.05	reference	0.05	reference	0.05
	Present	21 (13.8)	57 (21.5)		1.71 (0.99 – 2.95)		1.71 (0.99 – 2.95)	
Vaginal bleeding after sex	Not present	144 (94.7)	251 (94.7)	1.0	reference	1.0	reference	1.0
	Present	8 (5.3)	14 (5.3)		1.00 (0.41 – 2.45)		0.99 (0.40 – 2.42)	
Vaginal sores	Not present	146 (96.1)	244 (92.1)	0.1	reference	0.1	reference	0.1
	Present	6 (3.9)	21 (7.9)		2.09 (0.83 – 5.31)		2.10 (0.83 – 5.31)	
Dysuria	Not present	132 (86.8)	231 (87.2)	0.9	reference	0.9	reference	0.9
	Present	20 (13.2)	34 (12.8)		0.97 (0.54 – 1.76)		0.95 (0.52 – 1.75)	
Hematuria ^†††^	Not present	150 (98.7)	250 (94.3)	0.03	reference	0.05	reference	0.05
	Present	2 (1.3)	15 (5.7)		4.49 (1.02 – 40.99)		4.44 (1.00 – 40.63)	
Abdominal pain	Not present	108 (71.0)	192 (72.5)	0.8	reference	0.8	reference	0.7
	Present	44 (28.9)	73 (27.5)		0.93 (0.60 – 1.45)		0.92 (0.59 – 1.44)	
Delay in conception ^‡^	No	112 (91.8)	193 (91.9)	1.0	reference	1.0	reference	1.0
	Yes	10 (8.2)	17 (8.1)		0.99 (0.44 – 2.22)		1.01 (0.45 – 2.92)	

*Chi squared p-value **Adjusted for age and district of residence †Likelihood ratio test p-value ††(Reviewer 1) Declined to answer (n=33) and not applicable (n=95) are not included in the table (54 visual FGS not detected; 37 FGS; 37 missing) †††Odds Ratios and p-values obtained through exact logistic regression in both crude and adjusted analyses ‡(Reviewer 2) Declined to answer (n=33) and missing (n=95) are not included in the table; (30 visual FGS not detected; 55 FGS detected; 43 missing)

## Discussion

Diagnostics for neglected tropical diseases should be accurate, accessible, and affordable, with specimen collection that is easy ^26^. Making a diagnosis of FGS is challenging as there is currently not a widely accessible, sensitive and non-invasive reference standard for either diagnosis or screening which confirms *Schistosoma* genital involvement at the point-of-care. In a 2010 expert-led consensus meeting, visual imaging of the vagina and cervix with photocolposcopic methods was proposed as an adequate reference standard for FGS visual diagnosis ^27^. Imaging is currently the only widely available point-of-care diagnostic tool for FGS diagnosis outside of the research setting and the BILHIV study sought to use hand-held colposcopy to enable community-based FGS diagnosis ^9^. Visual imaging can be useful in the assessment of *Schistosoma*-related morbidity, praziquantel treatment response, and defining the natural history of visual-FGS. Additionally, hand-held and traditional colposcopy have the logistical advantage that they can be integrated with existing cervical cancer screening programmes ^28^. However, visual imaging has important limitations. Firstly, interpretation of visual imaging is subjective. Secondly, visual imaging lacks specificity as the characteristic sandy patches can also be associated with STI and the abnormal blood vessels can also be associated with cervical precancer ^3^. This study shows “slight” agreement between senior, highly experienced expert reviewers, highlighting the imperfect nature of human expert review of images for FGS. 

Visual FGS-diagnosis is a widely accepted diagnostic tool for evaluating *Schistosoma*-associated genital morbidity. However, visual-FGS screening is often centralized in settings with access to traditional colposcopy and is invasive, requiring vaginal speculum insertion and trained medical professionals (physicians, nurses, or midwives) to visualize the cervix and vagina at high resolution ^9^. Additionally, visual-FGS diagnosis requires a full inspection of the mucosal surfaces of the vagina and cervix. If metal specula are used, post-examination autoclaving and appropriate disinfection further constrains the settings in which this diagnostic strategy can be seamlessly implemented. Disposable specula have risks and benefits. While hygienic and convenient, disposable plastic specula may not be sturdy enough when rotated to inspect the anterior and posterior vaginal walls and may contribute to missed visual-FGS diagnoses ^6^. A good light source is needed for optimal cervicovaginal visualization ^6^, as well as a device which can provide adequate magnification, ideally a colposcope, hand-held colposcope, or digital camera ^8^. Thus, colposcopy, whether hand-held or traditional, for visual-FGS diagnosis is not readily scalable for use as a population-based screening technique.

In this current work, without complete STI and HPV testing or cervicovaginal biopsy on each participant, it is challenging to assess the significance of the sandy patches and abnormal blood vessels identified by the clinical expert reviewers. Notably, researchers in Tanzania performed macroscopic cervicovaginal examinations comparing *S. haematobium* endemic and non-endemic areas, finding 75% of participants in endemic areas had cervical lesions (including sandy patches, edema, erosions and petechiae) compared with 36% of women in non-endemic areas (although their travel and medical history were not described) ^29^. The Tanzanian study illustrates the limited specificity of visual techniques, since one-third of the women had cervical lesions in communities where *S. haematobium* is not endemic.

Other diagnostic approaches such as PCR-based methods, have been implemented in research settings but are not yet field-deployable ^9^. Antigen, antibody, and pathogen-based diagnostics (such as microscopy) are useful diagnostic adjuncts for *Schistosoma* infection, but do not confirm the involvement of genital tissue. Future diagnostic algorithms may be optimized by first performing a microbiologic *S. haematobium* diagnosis prior to performing screening for genital involvement ^9^. Promising pathogen detection strategies that can be implemented at the point-of-care include isothermal DNA amplification methods ^30, 31^. These field-deployable molecular assays should be further developed for use at the point-of-care to identify *Schistosoma* DNA in self-collected genital swabs ^31^.

Our study did not show a consistent association between expert diagnosis of visual-FGS and abdominal, genitourinary and reproductive symptoms. Reviewer 1’s evaluation suggested an association between self-reported delay in conception and visual-FGS and Reviewer 2’s evaluation suggested a weak association with hematuria and dyspareunia in participants with visual-FGS. A retrospective study from Tanzania evaluating histopathology reported tubal schistosomiasis in 4 patients reporting with infertility ^32^ and a cross-sectional study from Zimbabwe found strong evidence that the presence of *S. haematobium* in pap smear was associated with infertility in women aged 20 – 49 years, after adjusting for age and HIV status ^5^. While alluring to consider the association of delayed conception identified by one reviewer with visual-FGS in isolation, the association would have been strengthened by consistency of the findings across reviewers. Additionally, in interpreting this result, it is important to consider the possibility of a type 1 error when large numbers of statistical tests are performed.

Previous work on visual-FGS has compared visual imaging to other diagnostic standards ^33^ or have used computerized algorithms ^34, 35^, or a combination of human reviewers and a digital gridding technique to evaluate visual-FGS ^7^. A recent Madagascan study utilized human reviewers together with a digital image gridding technique to review images of women with known FGS-associated clinical lesions and found a Fleiss kappa of 0.55 (“moderate” agreement) for detecting rubbery papules. Reviewers in that study achieved a higher agreement than that described in our study, potentially by undergoing an initial consensus rating exercise to reach agreement on uniform rating of images. Our approach in the BILHIV study illustrates a real-world scenario where expert reviewers may not necessarily have the opportunity for consensus agreement prior to consultation. This is the first study to assess the agreement of human expert reviewers for diagnosing visual-FGS with hand-held colposcopy, where both reviewers were blinded to the participants’ FGS and *Schistosoma* diagnostic status. In this study, both expert reviewers are experienced clinical Professors who have expertise in diagnosing FGS in endemic settings and contributed as authors to the 2015 WHO FGS Pocket Atlas ^16^.

While our approach is unique, this work has some limitations. The prevalence of urinary schistosomiasis and PCR-FGS were low, thus limiting precision in effect sizes and power to detect association when comparing PCR-FGS and urinary schistosomasis with visual-FGS. Additionally, the urban setting, relatively narrow age range of the participants and low urinary *S. haematobium* prevalence may limit generalizability. Future additional work in a setting with higher schistosomiasis prevalence would be needed to definitively exclude an association between symptoms, standard *Schistosoma,* and FGS diagnostics and visual-FGS. To replicate real-world conditions, standardized equipment on which to perform image review was not provided to reviewers. Thus, we cannot exclude that differences in color, brightness, contrast, or saturation of images on the reviewers’ computers contributed to differences between reviewers. Additionally, future work could incorporate artificial intelligence, such as computer algorithms to detect the characteristic color change caused by involvement of the genital mucosa with FGS ^35^ or the use of digital gridding techniques ^7^. Additionally, a initial consensus rating exercise could be incorporated into future work with human expert review for FGS-associated lesions. The presence or absence of the specific FGS lesion (sandy patch, rubbery papule, abnormal blood vessels) was not consistently documented along with the presence or absence of visual-FGS, limiting analysis by lesion type. Study participants self-reported their time-to-conception status, thus results may be subject to recall bias. STI testing was only performed on a subset of the study population and visual inspection with acetic acid data were not obtained within the BILHIV study, thus data on these variables are incomplete ^18^. Without complete STI and HPV testing or cervicovaginal biopsy on each participant, it is challenging to assess the significance of the sandy patches and abnormal blood vessels identified by the clinical expert reviewers. Thus, we cannot exclude residual or unmeasured confounding.

In conclusion, with only “slight” agreement between experienced expert reviewers who identified visual-FGS from digital images obtained during point-of-care colposcopy, we suggest caution when visual imaging is used as a stand-alone FGS diagnostic. While we await field-deployable molecular methods to supplement FGS diagnosis, further studies could evaluate if combining colposcopy for visual-FGS with point-of-care STI diagnostics might improve specificity. Our findings highlight the imperfect nature and challenges of visual diagnosis of FGS as a research and clinical endpoint and is a call to action for improved point-of-care diagnostics and diagnostic pathways for female genital schistosomiasis.
