# Supplementary material for: Visual diagnosis of female genital schistosomiasis in Zambian women from hand-held colposcopy: agreement of expert image review and association with clinical symptoms
Source: Wellcome Open Res. 2023 May 4;8:14. Originally published 2023 Jan 10. [Version 2] doi: 10.12688/wellcomeopenres.18737.2 (PMC9971661; doi:10.12688/wellcomeopenres.18737.2)
Supplement: Peer review history

# Peer review: Reviewer response for version 2

**DOI:** 10.21956/wellcomeopenres.21450.r56976

**Published:** 2023-07-03

**Authors:** Soledad Colombe

**Affiliations:** 1 Department of Public Health, Institute of Tropical Medicine, Antwerp, Belgium

I thank the authors for the changes brought to the manuscript. I have no further comments.

Is the work clearly and accurately presented and does it cite the current literature?

Yes

If applicable, is the statistical analysis and its interpretation appropriate?

Yes

Are all the source data underlying the results available to ensure full reproducibility?

Yes

Is the study design appropriate and is the work technically sound?

Yes

Are the conclusions drawn adequately supported by the results?

Yes

Are sufficient details of methods and analysis provided to allow replication by others?

Yes

Reviewer Expertise:

Infectious Diseases ; Epidemiology

I confirm that I have read this submission and believe that I have an appropriate level of expertise to confirm that it is of an acceptable scientific standard.

# Peer review: Reviewer response for version 2

**DOI:** 10.21956/wellcomeopenres.21450.r56977

**Published:** 2023-06-06

**Authors:** Vanessa Christinet

**Affiliations:** 1 ASCRES, Lausanne, Switzerland

Thank you for the answers.

Is the work clearly and accurately presented and does it cite the current literature?

Yes

If applicable, is the statistical analysis and its interpretation appropriate?

Partly

Are all the source data underlying the results available to ensure full reproducibility?

Yes

Is the study design appropriate and is the work technically sound?

Yes

Are the conclusions drawn adequately supported by the results?

Partly

Are sufficient details of methods and analysis provided to allow replication by others?

Partly

Reviewer Expertise:

Sexual health, HIV, communicable disease, tropical medicine, public health, epidemiology.

I confirm that I have read this submission and believe that I have an appropriate level of expertise to confirm that it is of an acceptable scientific standard.

# Peer review: Reviewer response for version 2

**DOI:** 10.21956/wellcomeopenres.21450.r56974

**Published:** 2023-05-16

**Authors:** Louise Arenholt

**Affiliations:** 1 Department of Clinical Medicine, Aalborg University, Aalborg, Denmark; 2 Department of Obstetrics and Gynecology and Centre for Clinical Research, North Denmark Regional Hospital, Hjørring, Denmark

I find the response and revision sufficient. I therefore recommend the manuscript for indexing.

Is the work clearly and accurately presented and does it cite the current literature?

Yes

If applicable, is the statistical analysis and its interpretation appropriate?

Yes

Are all the source data underlying the results available to ensure full reproducibility?

Partly

Is the study design appropriate and is the work technically sound?

Yes

Are the conclusions drawn adequately supported by the results?

Yes

Are sufficient details of methods and analysis provided to allow replication by others?

Partly

Reviewer Expertise:

Gynecology and obstetrics. Urogynecology.

I confirm that I have read this submission and believe that I have an appropriate level of expertise to confirm that it is of an acceptable scientific standard.

# Peer review: Reviewer response for version 1

**DOI:** 10.21956/wellcomeopenres.20776.r54085

**Published:** 2023-02-21

**Authors:** Soledad Colombe

**Affiliations:** 1 Department of Public Health, Institute of Tropical Medicine, Antwerp, Belgium

I thank the editor and authors for the opportunity to review this article, which brings to light relevant information for those working on female genital schistosomiasis. In this study, the authors tested the validity of the recommended visual diagnosis of FGS by assessing the concordance in visual diagnosis from two independent senior physicians. They found very low agreement between the independent reviewers, highlighting the complexity of using visual imaging for diagnosis of female genital schistosomiasis. The article was overall very sound and well written. I have made a few comments below on both format and scientific content, which I hope will be useful to the authors.

Comments on format:

- Please define CVL in the abstract before using the accronym.
- I would quickly define in the abstract what is meant by “expert reviewers”, and would also define it sooner in the methods in the main text.

Comments on scientific content:

 I am missing more in-depth description and discussion of the discordance in visual diagnosis between the two expert reviewers.

- a) I noticed that the discordance was already quite large for the number of images that could not be interpreted (36 vs 106). What does “images inaccessible” in Figure 1 mean? Could you explain this discordance?
- b) Regarding the discordance in visual diagnosis of FGS: I understand that the presence or absence of a specific FGS lesion was not systematically recorded but did you notice a systematicity in the discordance? Would this discordance have been solved in the majority of cases after discussions between the two expert reviewers?   
- c) Does the conclusion of “diagnostic uncertain” (n=23) of reviewer 1 overlap with the images discarded by reviewer 2 for “poor cervical visibility”?
- d) I would suggest to add pictures, if allowed by the journal, of images leading to concordant positive, concordant negative and discordant diagnoses.

It seems that in a number of participants, visual-FGS was “positive” whereas PCR/CAA/Microscopy were negative. In addition to the fact that the lesions thought during visual inspection can be non-specific, could it also be that visual-FGS in some cases is rather the sign of past infection? And in that case can the authors discuss the added-value of visual-FGS diagnostic?

Is the work clearly and accurately presented and does it cite the current literature?

Yes

If applicable, is the statistical analysis and its interpretation appropriate?

Yes

Are all the source data underlying the results available to ensure full reproducibility?

Yes

Is the study design appropriate and is the work technically sound?

Yes

Are the conclusions drawn adequately supported by the results?

Yes

Are sufficient details of methods and analysis provided to allow replication by others?

Yes

Reviewer Expertise:

Infectious Diseases ; Epidemiology

I confirm that I have read this submission and believe that I have an appropriate level of expertise to confirm that it is of an acceptable scientific standard, however I have significant reservations, as outlined above.

# Response

**Published:** 2023-04-05

**Authors:** Amy Sturt

**Affiliations:** Veterans Affairs Medical Center Palo Alto, USA

"Comments on format: -please define CVL in the abstract before using the acronym *Thank you for noticing this, this has been amended.*   -I would quickly define in the abstract what is meant by expert reviewers and would also define it sooner in the methods in the main text."

*Thank you for this suggestion. We inserted “senior physicians who have training and expertise in FGS and standard colposcopy served as expert reviewers” in the abstract at line 64. As you have suggested, we also included an enhanced definition of the expert reviewers in the methods at lines 152-153.*  

 "Comments on the scientific section: I am missing a more in-depth description and discussion of the discordance in visual diagnosis between the two expert reviewers. -a) I noticed that the discordance was already quite large for the number of images that could not be interpreted (36 vs 106). What does images inaccessible in Figure 1 mean? Could you explain this discordance?"   

*Thank you for the opportunity to clarify. In lines 156-157 we explain that, at their discretion, expert reviewers could exclude images that they felt could not be evaluated due to technical issues, image quality, or limited cervical visualization. This number was 36 for Reviewer 1 and 106 for Reviewer 2. Reviewer 1 had difficulty opening 10 of the images. In Figure 1 ‘images inaccessible’ has been changed to “technical difficulty opening images”.*  

 "-b) Regarding the discordance in visual diagnosis of FGS: I understand that the presence or absence of a specific FGS lesion was not systematically recorded but did you notice a systematicity in the discordance? Would this discordance have been solved in the majority of cases after discussions between the two expert reviewers?"   

*We did not observe a systematicity in the discordance. It is difficult to say whether a discussion between experts could have resolved the discordance. We did not include an initial consensus rating agreement in our study as we wanted to simulate real-world conditions in our study, where consultant physicians may not have time to perform a consensus review on a large number of images. We did note in the discussion at lines 407-408 that an initial consensus rating exercise could be incorporated into future work with human expert review for FGS-associated lesions.*  

 "-c) Does the conclusion of “diagnostic uncertain” (n=23) of reviewer 1 overlap with the images discarded by reviewer 2 for “poor cervical visibility”?"   

*Thank you for this question. The images discarded by Reviewer 2 seem to be rejected at random as there were diagnoses of FGS negative, sandy patches and abnormal blood vessels among the discarded images. Within these images there were a minority (n=6) that were also rejected by Reviewer 1. *  

 "-d) I would suggest to add pictures, if allowed by the journal, of images leading to concordant positive, concordant negative and discordant diagnoses."   

*Wellcome Open Research specifies that “Any photographs must be accompanied by written consent to publish from the individuals involved”. We did not obtain consent from the participants to publish cervicovaginal images* so unfortunately cannot include these.  

 "It seems that in a number of participants, visual-FGS was “positive” whereas PCR/CAA/miscropscopy were negative. In addition to the fact that lesions though during the visual inspection can be non-specific, could it also be that visual-FGS in some cases is rather the sign of past-infection? And in that case can the authors discuss the added value of visual-FGS diagnostic?"   

*Thank you for this observation. We agree that it is possible that visual-FGS may be representative of past infection and agree that in this setting, its utility as a FGS diagnostic is limited. We feel strongly that the way forward in FGS diagnosis is through the further development, implementation, and scale-up of molecular diagnostics for current FGS, as outlined in lines 421-423.*

# Peer review: Reviewer response for version 1

**DOI:** 10.21956/wellcomeopenres.20776.r54091

**Published:** 2023-02-21

**Authors:** Susannah Colt

**Affiliations:** 1 Center for International Health Research, Rhode Island Hospital, Providence, RI, USA

Summary of the article:

 Female genital schistosomiasis (FGS) is an underreported and debilitating illness affecting women and girls and is widespread among *Schistosoma haematobium*-endemic regions throughout Africa. There are significant challenges to accurate FGS diagnosis relating to lack of a consensus method, equipment availability, technical expertise, and stigma surrounding women’s reproductive health. While the proposed reference-standard for FGS diagnosis is visual inspection by colposcopy (visual-FGS), this method is subjective to expert interpretation and can be further confounded by some sexually transmitted infections. The authors report secondary findings of the cross-sectional bilharzia and HIV (BILHIV) study, in which, N=527 women from Zambia were enrolled; provided urine, cervical and vaginal self-sampled tissue swabs; and underwent clinical cervicovaginal lavage sampling and portable colposcope examination and imaging. The primary aim was to evaluate the agreement of visual-FGS by two independent experts, and the secondary aims were to compare visual-FGS with other FGS and *S. haematobium* diagnostic techniques as well as self-reported FGS sequelae. While the proportion of FGS cases—by any diagnostic method—was relatively low in this cohort, the authors report only slight agreement between the two visual-FGS experts. These results further emphasize the challenges associated with FGS diagnosis and the need for more reliable and accessible diagnostic methods. This study provides an important contribution to the FGS literature and calls attention to barriers to providing reproductive health care for African women.

Introduction:

1. The authors should provide some background epidemiological info on burden of FGS among women and girls in Africa, associated morbidities (relating to both physical and mental health as some of these are a feature of the secondary analysis), and emphasize that cases are underdiagnosed due to a myriad of reasons (equipment, expertise, access to women's health services, etc). If available, include any info on the burden of urogenital schistosomiasis in Zambia or offer context for why Zambia was chosen as the study site. 
2. Please describe techniques for identifying FGS in the introduction (biopsy of genital tissue, visual inspection by colposcopy, PCR performed on cervicovaginal lavage material) and include the advantages and disadvantages of each. 
3. It would be helpful to clarify in the introduction that this investigation reports findings of the BILHIV cohort study.
4. Suggestion to state the primary and two secondary objectives of the present study in the introduction section.

Methods:

1. The study benefits from the selection of two highly qualified visual-FGS experts, inclusion of advanced techniques in FGS diagnoses including qPCR of CVL material, and application of the Mobile ODT hand-held colposcope which is one of the devices recommended for FGS diagnosis in schistosomiasis-endemic areas in a 2021 systematic review of hand-held colposcopy equipment.
2. It would be helpful for the authors to clarify which participants were included for STI evaluation and the criteria for how this subset was selected.
3. Please provide detail about the regression model building approach and explanation for the inclusion for age or age and district as covariates. Have the authors considered additional potential confounding factors, such as co-infection with HIV or other STIs, other co-morbidities, sexual history variables, or pregnancy history?

Results:

1. There is a typo in the word “assessment” at the end of the "Visual FGS and Schistosoma laboratory tests” paragraph.
2. It would be interesting for authors to report the agreement between tissue-specific qPCR results (cervical swab, vaginal swab, and CVL).

Discussion:

1. A strong case is presented for the disadvantages of visual-FGS diagnosis.
2. The authors discuss the limited feasibility for PCR-based diagnosis in schistosomiasis-endemic areas and make reference to promising developments in Loop-Mediated Isothermal Amplification (LAMP) methods, which offer added accessibility in resource-limited settings. The authors might consider incorporating discussion or reference to demonstrated LAMP sensitivity for *S. japonicum* and *S. haematobium* (Wang *et al.* 2011 ^1^ and Gandasegui *et al.*, 2015 ^2^).

Is the work clearly and accurately presented and does it cite the current literature?

Yes

If applicable, is the statistical analysis and its interpretation appropriate?

Yes

Are all the source data underlying the results available to ensure full reproducibility?

Yes

Is the study design appropriate and is the work technically sound?

Yes

Are the conclusions drawn adequately supported by the results?

Yes

Are sufficient details of methods and analysis provided to allow replication by others?

Yes

Reviewer Expertise:

Epidemiology and biostatistics; schistosomiasis; infectious disease immunology

I confirm that I have read this submission and believe that I have an appropriate level of expertise to confirm that it is of an acceptable scientific standard.

## References

[1] : Application of DNA-based diagnostics in detection of schistosomal DNA in early infection and after drug treatment. *Parasit Vectors* .2011;4: 10.1186/1756-3305-4-164 164 10.1186/1756-3305-4-164 21864384PMC3177784

[2] : The Rapid-Heat LAMPellet Method: A Potential Diagnostic Method for Human Urogenital Schistosomiasis. *PLoS Negl Trop Dis* .2015;9(7) : 10.1371/journal.pntd.0003963 e0003963 10.1371/journal.pntd.0003963 26230990PMC4521856

# Response

**Published:** 2023-04-05

**Authors:** Amy Sturt

**Affiliations:** Veterans Affairs Medical Center Palo Alto, USA

"Introduction – The authors should provide some background epidemiological info on burden of FGS among women and girls in Africa, associated morbidities (relating to both physical and mental health as some of these are a feature of the secondary analysis), and emphasize that cases are underdiagnosed due to a myriad of reasons (equipment, expertise, access to womens health services, etc). If available, include any info on burden of urogenital schistosomiasis in Zambia or offer context for why Zambia was chosen as a study site." *Thank you for your input. In lines 89-90 we have added some associated morbidities as you have suggested. Schistosomiasis is endemic in Zambia as outlined in a recent systematic review:* *https://pubmed.ncbi.nlm.nih.gov/29706131/* *. A sentence regarding the S. haematobium prevalence in Zambia has been inserted at lines 122 – 124, as you have suggested. The BILHIV study was nested within the HPTN 071 (PopART) trial, which was enrolling in Zambia, providing an opportunity to further evaluate urogenital schistosomiasis in this region. We have added additional information about HPTN in Zambia in lines 124-125. We agree 100% that FGS is underdiagnosed due to the reasons you have outlined above. We had a more complete discussion on the challenges of making a FGS diagnosis in an initial draft of the manuscript. Ultimately, we removed these details as we felt the detail detracted from the main focus of this manuscript - visual FGS. We discuss the challenges inherent in making a FGS diagnosis in the main BILHIV manuscript, which we have now referenced earlier at line 96. *  

 "2) Please describe techniques for identifying FGS in the introduction (biopsy of genital tissue, visual inspection by colposcopy, PCR performed on cervicovaginal lavage material) and include the advantages and disadvantages of each."   

*Thank you for this suggestion, we initially had a more complete discussion of FGS methods in an earlier draft of the manuscript, but felt the depth (and length) detracted from the main focus. Instead we have summarized the challenges of making a FGS diagnosis in lines 98 -99 by saying “Confirming a diagnosis of FGS is challenging as there is not a widely accepted diagnostic reference standard for research, diagnosis, and screening.” Additionally, we have included a more complete review of the advantages and disadvantages of the available FGS screening methods in the discussion.*  

 "3) It would be helpful to clarify in the introduction that this investigation reports findings of the BILHIV cohort study."   

*Thank you for this input, this is now clearly stated in the introduction at line 113.*  

 "4) Suggestion to state the primary and two secondary objectives of the present study in the introduction section."   

*Thank you for this suggestion. The primary and secondary objectives of the analysis are now stated in the introduction from lines 114 – 117.*  

 "Methods: 1.The study benefits from the selection of two highly qualified visual-FGS experts, inclusion of advanced techniques in FGS diagnosis including qPCR of CVL material, and application of the Mobile ODT hand-held colposcope which is one of the devices recommended for FGS diagnosis in schistosomiasis-endemic areas in a 2021 systematic review of hand-held colposcopy equipment."   

*We thank the reviewer from highlighting this strength.*  

 "2.It would be helpful for authors to clarify which participants were included for STI evaluation and the criteria for how this subset was selected."   

*Thank you for the opportunity to provide additional information. We have added some information about this subset and their selection criteria in the Methods section “other infections” at lines 205-211.*  

 "3.Please provide detail about the regression model building approach and explanation for the inclusion of age or age and district as covariates. Have the authors considered additional potential confounding factors such as co-infection with HIV or other STIs, other co-morbidities, sexual history variables or pregnancy history?"   

*The regression model was built after construction of causal conceptual frameworks with age and district included *a priori *. District of residence may potentially be a surrogate for socioeconomic status and/or probability of schistosomiasis exposure (one district was closer to a local river). Additional variables (including prior history of pregnancy) were included in the model in a step-wise fashion and retained if they produced a 10% change in Odds Ratio. No additional variables produced a change in OR and thus were not retained in the model. Since HIV has not been consistently associated with FGS across studies, this variable was not included. STI and cervical pre-cancer were considered for inclusion in the model. Given the large amount of missing data in the STI and cervical pre-cancer variables, these were not included in the regression analysis. We also investigated the effect of adjusting for HIV status and found it made no difference to our findings.  STI and cervical cancer testing were performed on a subset of well under half of the participants. Including these variables as potential confounders in the regression would have led to further data sparsity. Despite this, we conducted an additional exploratory analysis where we controlled for STI (and separately, VIA) when assessing the association between FGS diagnosis and symptoms, and compared the ORs with those from adjusting for only age and district, restricted to participants who had STI (or VIA) information available (i.e. in order to compare ORs on the same subset of participants). We found no suggestion that STI or VIA act as confounders, and, as already noted, power to detect associations was substantially reduced in this analysis. *  

 "Results: 1.There is a typo in the word assessment at the end of the “visual FGS and schistosoma laboratory tests paragraph”." 

*Thank you, this has been amended.*  

 "2.It would be interesting for authors to report the agreement between tissue specific qPCR results." 

*Thank you for this suggestion, the full details regarding agreement between tissue-specific qPCR results* *are reported in a previous manuscript. We have clarified that further details regarding the operating characteristics of these diagnostics have been previously described and have added in lines at 284-285 referring the reader to the previous manuscript. Full details can be found at* *https://journals.plos.org/plosntds/article?id=10.1371/journal.pntd.0008337*

 "Discussion: 1.A strong case is presented for the disadvanges of visual-FGS diagnosis." 

*Thank you for this feedback.*

 "2.The authors discuss the limited feasibility for PCR-based diagnosis in schistosomiasis-endemic areas and make a reference to promising developments in LAMP methods, which offer added accessibility in resource limited settings. The atuhors might consider incorporating discussion or reference to demonstrated LAMP sensitivity for S. japonicum and S. haematobium (wang 2011- Parasit Vectors and Gandasegui 2015 - PNTD)." 

*Thank you for this helpful reference. Gandasegui 2015 was entered at line 354.*

# Peer review: Reviewer response for version 1

**DOI:** 10.21956/wellcomeopenres.20776.r54089

**Published:** 2023-02-08

**Authors:** Vanessa Christinet

**Affiliations:** 1 ASCRES, Lausanne, Switzerland

Thank you for inviting me to review this article on a comparative analysis of visual diagnosis of FGS between two experts in visual diagnosis of cervical images. The authors chose to explore the relationship between the diagnosis based on image analysis of the two reviewers and several objective measurements of S. Heamatobium infection diagnosis and also with symptoms described by the patients.

 This study and its results are very relevant and interesting as there is little produced in this field of research. It is indeed very important to evaluate diagnosis by visual inspection of the cervix as this is the recommended method of reference but there is a lack of data on the reliability of the diagnosis.

 As this study is nested within the HPTN071 study, I would recommend to briefly describe the HPTN071 study in a single sentence, even if it has already been described in other publications, in order to fully understand the profile of the participants and the general context of the study.

 Indeed, the question of HIV diagnosis and the level of immunosuppression (if known) should be addressed as it is certainly an important confounding factor for the interpretation of the visualized lesions and could induce an important heterogeneity of cervical lesions making them more difficult to interpret (association between HIV and FGS, association between HIV and other STIs, association between HIV-HPV-dysplasia).

 In the description of the method, it is stated that STIs were not screened for POC but later in the text it is written that a whole panel of STIs was tested. Considering that the authors state that microbiological STI diagnosis can be a confounding factor in the diagnosis and symptoms associated with FGS, it seems important to me that they are taken into account in the analysis. As this is not the case here, it would be relevant to justify why.

 Cervical cancer screening with visualization of dysplasia is described in the methodology but is not included in the comparative analysis between the two reviewers despite the fact that it is described as a confounder in terms of vascularization.

 In the statistical analysis section it is described that the OR was adjusted for age. I would like the authors to describe the rationale for this adjustment and why other confounding factors were not included in this adjustment (STI, HIV, cervical dysplasia). Age seems to me to be relevant for the adjustment of the association with delay of conception, but I do not understand the interest of using it for the other variables (it should be noted that the unadjusted and adjusted ORs are not very different, which speaks to a moderate interest for the inclusion of this adjustment factor).

 In the method, additional detail on the number of photos per patient sent to the reviewers would be an enhancement as this could give more information on what they based their evaluation on. It would also be interesting to have more detailed information on the quality of the images to help understand the discrepancy between the two reviewers' analysis. Was a precise description of the lesions made by each of the reviewers which would allow a better understanding of the level of disagreement?

 It is noteworthy that although not reaching statistical significance, both reviewers consistently identified a greater proportion of FGS lesions in individuals with objective markers of schistosome infection. If the sample size had been larger, statistical significance would probably have been reached for some variables. If the data are available, I think it would be very interesting to exploit the STI diagnoses here to see how they probably contributed to the heterogeneity of the analysis.

 I find the presentation of the results not very easy to understand. The authors refer several times to the non-association without describing precisely the significance of the analysis which is actually the lack of a statistically significant difference between patients diagnosed by positive or negative visual inspection by the reviewers in relation to objective parameters of schistosome infection. Again, it should be noted that even if the differences are not statistically significant, the ORs are still positive in favour of the objective measurements of schistosomiasis. I suggest rewording the text below Table 3.

 It is interesting to note that the symptom with the highest OR for both reviewers is for the association with haematuria, which is the most specific symptom of Schistosoma Heamatobium infection.

Is the work clearly and accurately presented and does it cite the current literature?

Yes

If applicable, is the statistical analysis and its interpretation appropriate?

Partly

Are all the source data underlying the results available to ensure full reproducibility?

Yes

Is the study design appropriate and is the work technically sound?

Yes

Are the conclusions drawn adequately supported by the results?

Partly

Are sufficient details of methods and analysis provided to allow replication by others?

Partly

Reviewer Expertise:

Sexual health, HIV, communicable disease, tropical medicine, public health, epidemiology.

I confirm that I have read this submission and believe that I have an appropriate level of expertise to confirm that it is of an acceptable scientific standard, however I have significant reservations, as outlined above.

# Response

**Published:** 2023-04-05

**Authors:** Amy Sturt

**Affiliations:** Veterans Affairs Medical Center Palo Alto, USA

"As this study is nested within the HPTN study, I would recommend to briefly describe the HPTN study in a single sentence, even if it has already been described in other publications, in order to fully understand the profile of the participants and the general context of the study." *Thank you for this feedback. The HPTN 071 (PopART) trial was a cluster randomized trial assessing the impact of an HIV-1 combination prevention package including “universal testing and treatment”. We have added this information in lines 124-125.*  

 "Indeed, the question of HIV diagnosis and the level of immunosuppression (if known) should be addressed as it is certainly a confounding factor for the interpretation of the visualized lesions and could induce an important heterogeneity of cervical lesions making them more difficult to interpret (association between HIV and FGS, association between HIV and other STI, association between HIV-HPV-dysplasia)."   

*Data were not collected from participants regarding CD4 count or HIV viral load for this study. We agree that HIV-1 status should be assessed in future manuscripts which would evaluate associations between HIV and STI or HIV, HPV and cervical dysplasia. For the primary outcome of this study (agreement between expert reviewers), we acknowledge that HIV could induce heterogeneity in the lesions. However since the participants’ HIV-1 diagnosis in this study was concordant between reviewers, and our primary purpose was to describe agreement between expert reviewers (irrespective of other participant characteristics), we believe that it is appropriate to describe agreement in visual-FGS diagnosis without adjustment for HIV or other factors that could influence the nature of the lesions. *  

 "In the description of the method, it is stated that STIs were not screened for POC but later in the test it is written that a whole panel of STI was tested. Considering that the authors state that microbiological STI diagnosis can be a confounding factor in the diagnosis and symptoms associated with FGS, it seems important to me that they are taken into account in the analysis. As this is not the case here it would be relevant to justify why."   

*Thank you for this enquiry. The expert image review was performed and completed prior to STI testing. Thus, STI test results were not available at the time of expert image review to supplement the reviewers’ findings. The primary aim of this study was to evaluate the agreement of human expert reviewers for the diagnosis of visual-FGS. Due to study design and budgetary constraints, STI testing was only performed on a subset of study participants. This has been further clarified in the manuscript from lines 205-211. Overall, 40% (211/527) of participants in this study had STI data available, limiting power to evaluate associations between STI, visual-FGS, and symptoms. Thus the numbers were not large enough to meaningfully control for STI. Despite this, we have conducted an exploratory analysis where we additionally controlled for STI when assessing the association between FGS diagnosis and symptoms, restricted to the sub-group of participants who had STI information available (i.e. in order to assess the potential confounding affect of STI among the same subset of participants, comparing like with like). In this exploratory analysis we found no suggestion that STI acts as a confounder.*  

 "Cervical cancer screening with visualization of dysplasia is described in the methodology but is not included in the comparative analysis between the two reviewers despite the fact that it is described as a confounder in terms of vascularization."   

*Thank you for this enquiry. Cervical cancer screening was not performed as part of the BILHIV study. Cervical cancer screening results were extracted from participant medical records after expert image review was performed and completed. Thus, cervical pre-cancer status was not available at the time of expert image review to supplement the reviewers’ findings. For the primary outcome of this study (agreement between expert reviewers), we acknowledge that cervical pre-cancer could induce heterogeneity in the lesions. However since the participants’ cervical pre-cancer diagnosis in this study was concordant between reviewers, and our primary purpose was to describe agreement between expert reviewers (irrespective of other participant characteristics), we believe that it is appropriate to describe agreement in visual-FGS diagnosis without adjustment for cervical pre-cancer or other factors that could influence the nature of the lesions. *  

 "In the statistical analysis section it is described that the OR was adjusted for age. I would like the authors to describe the rationale for this adjustment and why other confounding factors were not included in the adjustment (STI, HIV, cervical dysplasia). Age seems to be to be relevant for the association with delay of conception but I do not understand the interest of using it for the other variables (it should be noted that the unadjusted and adjusted ORs are not different which speaks to a moderate interest for the inclusion of this adjustment factor)." *Thank you for the opportunity to clarify the statistical methods. Age was prioritized as a confounder to include since it has been associated in the literature with both FGS and presence of symptoms. We also investigated the effect of adjusting for HIV status and found it made no difference to our findings.  As described above, STI and cervical cancer testing were performed on a subset of well under half of the participants. Including these variables as potential confounders in the regression would have led to further data sparsity. Despite this, we conducted an additional exploratory analysis where we controlled for STI (and separately, VIA) when assessing the association between FGS diagnosis and symptoms, restricted to the sub-group of participants who had VIA (or STI) information available (i.e. in order to assess the potential confounding affect of VIA among the same subset of participants, comparing like with like). In this exploratory analysis we found no suggestion that VIA acts as a confounder. As already noted, power to detect associations was reduced in this analysis.*  

 "In the method, additional data on the number of photos per patient sent to reviewers would be an enhancement as this could give more information on what they based their evaluation on. It would also be interesting to have more detailed information on the quality of the images to help understand the the discrepancy between the two reviewers’ analysis. Was a precise description of the lesions made by each of the reviewers which would allow a better understanding of the level of disagreement. It is noteworthy that although not reaching statistical significance, both reviewers consistently identified a greater proportion of FGS lesions in individuals with objective markers of schistoomiasis. If the sample size has been larger, statistical significance would probably be reached for some variables. If the data are available, I think it would be very interesting to exploit the STI diagnoses here to see how they probably contributed to the heterogeneity of the analysis."   

*Thank you for this question. Please see lines 149-153 where we have added in additional information about the standardized protocol used to evaluate each participant. The standardized protocol specified that trained midwives evaluated the cervix, anterior fornix, posterior fornix, left and right lateral cervix and vaginal walls for each participant and captured images of each location. In lines 154-155 we describe that each reviewer noted the presence or absence of one of four clinical manifestations associated with FGS. The reviewers did not note the location or further descriptive information regarding the clinical manifestations. It would have been ideal to have a larger sample size, however it is not possible to extrapolate our findings to what they might have been had the sample size been larger. Unfortunately, due to STI only being evaluated on a sub-set of participants, we are not able to adjust associations for STI among the full study population. However, as noted in our response above, in the subset of participants who did have STI information, we adjusted for STI when assessing associations between FGS-image positivity and symptoms and found that it made little difference to the odds ratios, in the subset of participants who had STI information available.*  

 "I find the presentation of the results not very easy to understand. The authors refer several times to the non-association without describing precisely the significance of the analysis which is actually the lack of a statistically significant difference between patients diagnosed by positive or negative visual inspection by the reviewers in relation to objective parameters of schistosome infection. Again it should be noted that even if the difference is not statistically significant, the ORs are still positive in favor of thee objective measurements of schistosomiasis. I suggest rewording the text below Table 3. It is interesting to note that the symptom with the highest OR for both reviewers is for the association with hematuria, which is the most specific symptoms of Shaematobium infection."     

*Thank you for this input and we apologize for any lack of clarity in the presentation of the results. In lines 292-295 we describe that vaginal discharge, vaginal bleeding after sex, the presence of external genital sores, dysuria and abdominal pain were not associated with the presence of visual FGS as diagnosed by either expert reviewer. In regards to the OR in Table 4 being in favor of an association between *Schistosoma *diagnostics and visual-FGS, the confidence intervals include 1, suggesting that there is not evidence of a difference between groups.* *Thank you, we agree this is an interesting finding.*

# Peer review: Reviewer response for version 1

**DOI:** 10.21956/wellcomeopenres.20776.r54092

**Published:** 2023-02-03

**Authors:** Daniela Fusco

**Affiliations:** 1 Infectious Disease Epidemiology, Bernhard Nocht Insitute for Tropical Medicine, Hamburg, Germany

The manuscript deals with a very relevant topic in need for urgent solutions. The methods and design are adequate for the study and the results are properly described. 

 Some minor recommendations to the manuscript:

- Title: In the title exclusively the aspect of the agreement of experts emerges, while in the results there is a long description of a regression analysis to associate factors (i.e. symptoms) to visual FGS. This aspect should emerge in the title. Additionally in the abstract it is stated that "The agreement of expert human reviewers for visual-FGS has not been previously described". In this view, the hand-held colposcopy doesn't play a central role in the study hence it should be considered to be removed from the title. 
- Methods: In the paragraph "Home and clinic-based sample collection" the type health care professionals performing the sampling should be specified. In the paragraphs "qPCR for detection of Schistosoma DNA" and "Other infections" samples storage conditions should be specified. 
- Discussion: As per in the title, in this statement "This study shows “slight” agreement between senior, highly experienced expert reviewers, highlighting the imperfect nature of human expert review of images obtained with hand-held colposcopy for FGS." the reader can have the feeling that the agreement might be different if another type of colposcope would be used. It would be advisable to reconsider the statement. From the reference 26 the statement "The Tanzanian study illustrates the limited specificity of visual techniques, since one-third of the women had cervical lesions in communities where S. haematobium is not endemic.” cannot be really deduced since, i.e., travel or medical history of women is not described. In this view this statement doesn't seem to reflect the message of the reference and it should be re-considered. The conclusion statement "...we suggest caution when visual imaging is used as a stand-alone FGS diagnostic." should bring some recommendations i.e. on how to interpret or obtain better results through colposcopy since, so far, colposcopy is still the diagnostic standard for the disease and alternatives, even if urgent and needed, are not really available. 

Is the work clearly and accurately presented and does it cite the current literature?

Yes

If applicable, is the statistical analysis and its interpretation appropriate?

Yes

Are all the source data underlying the results available to ensure full reproducibility?

Yes

Is the study design appropriate and is the work technically sound?

Yes

Are the conclusions drawn adequately supported by the results?

Partly

Are sufficient details of methods and analysis provided to allow replication by others?

Yes

Reviewer Expertise:

Public Health, schistosomiasis, FGS, women's health, diseases prevention, vaccinology

I confirm that I have read this submission and believe that I have an appropriate level of expertise to confirm that it is of an acceptable scientific standard.

# Response

**Published:** 2023-04-05

**Authors:** Amy Sturt

**Affiliations:** Veterans Affairs Medical Center Palo Alto, USA

"1)In the title exclusively the aspect of the agreement of experts emerges, while in the results there is a long description of regression analysis to associate factors (ie symptoms) to visual FGS. This aspect should emerge in the title. Additionally, in the abstract it is stated that “the agreement of expert reviewers for visual-FGS has not been previously described”. In this view the hand-held colposcopy doesn’t play a central role in the study hence it should be considered to be removed from the title."   

*Thank you for this input. We have added “and association with clinical symptoms” to the title to tie in the results from the regression analysis which evaluated the association of visual-FGS with clinical manifestations. We appreciate your perspective on omitting “hand-held colposcopy” from the title. Since hand held colposcopy is a unique and scalable technology, we feel it is important to highlight its use in the title.*  

 "2) Methods – In the paragraph “home and clinic based sample collection” the type health care professional performing the sampling should be specified. In the paragraphs “qPCR for detection of schistosoma DNA” and “other infections” sample storage conditions should be specified."   

*Thank you for the opportunity to clarify. At line 143 we explain that trained midwives performed sample collection. Additionally, all study specimens were stored at -80C. This has been clarified at line 193.*  

 "3) Discussion: As per the title, in this statement “This study shows slight agreement between senior, highly experienced expert reviewers, highlighting the imperfect nature of human expert review of images obtrained with hand-held colposcopy for FGS”. The reader can have the feeling that the agreement might be different if another type of colposcope would be used. It would be advisable to reconsider the statement. From the reference 26 the statement “the Tanzanian study illustrates the limited specificity of visual techniques since 1/3 of the women had cervical lesions in communities where S. haematobium is not endemic” cannot really be deduced since i.e. travel or medical history of women is not described. In this view this statement doesn’t seem to reflect the message of the reference and it should be reconsidered. The conclusion statement, “we suggest cauthion when visual imaging is used as a stand-alone FGS diagnostic” should bring some recommendations on how to interpret of obtain better results through colposcopy, since, so far, colposcopy is still the diagnostic standard for the disease and alternatives, even if urgent and needed, are not really available."   

*Thank you for your input on the discussion. As you have suggested, we have removed “obtained with hand-held colposcopy” from the referenced excerpt in the discussion and the abstract. Thank you for your perspective on the Tanzanian study (reference 26). On page 1210 of the referenced manuscripts the manuscript authors (Poggensee et al) note that “schistosomiasis is not endemic in this area” and used this group as their control group (against which to compare the participants who lived in areas endemic for schistosomiasis), and on page 1211 the authors refer to the participants who lived in non-endemic locations as “women living in schistosomiasis free areas”. However, it is true that travel and medical history of women is not described, and we now acknowledge this in the discussion. Thank you for the opportunity to provide further recommendations about how to obtain better results through colposcopy. This is difficult given the low specificity of visual imaging for FGS. Further studies are needed to evaluate if combining colposcopy for visual-FGS diagnosis with point-of-care STI testing might improve specificity. As you have suggested, this has been added to the manuscript’s discussion at lines 342-343.*

# Peer review: Reviewer response for version 1

**DOI:** 10.21956/wellcomeopenres.20776.r54086

**Published:** 2023-01-31

**Authors:** Alexander B. Odaibo

**Affiliations:** 1 Parasitology Research Unit, Department of Zoology, University of Ibadan, Ibadan, Nigeria

The authors evaluated the agreement of human expert reviewers on visual-FGS diagnosis with hand-held colposcopy. Two clinical experts in the diagnosis of FGS in endemic settings were engaged in the study. They assessed 527 women cervicovaginal colposcopy images independently. The study recorded a "slight" agreement between the expert reviewers of the digital images obtained during point-of-care colposcopy. The design was good and adequate but there is a need to improve on the details of some aspects of the methods to allow replication by others. 

1. Urine sample was collected during home visit - the authors should provide specific time of urine collection, as this is important in the diagnosis of schistosomiasis. Was the time of collection the same for all participants?
2. Up to 50 mL of urine was centrifuged and examined - the authors should provide the specific amount of urine centrifuged, and why 50 mL and not 10 mL as recommended by WHO (2022 ^1^), and for ease of comparison with other studies.
3. Figure 1. Flowsheet of cervicovaginal image review - the authors should explain what "Images inaccessible" means, particularly as it was among those who attended clinic.
4. The authors use the phrase "water use" and "water contact" interchangeably. I think "water contact" is best for this study.

Is the work clearly and accurately presented and does it cite the current literature?

Yes

If applicable, is the statistical analysis and its interpretation appropriate?

I cannot comment. A qualified statistician is required.

Are all the source data underlying the results available to ensure full reproducibility?

Yes

Is the study design appropriate and is the work technically sound?

Yes

Are the conclusions drawn adequately supported by the results?

Yes

Are sufficient details of methods and analysis provided to allow replication by others?

No

Reviewer Expertise:

Parasitologist/Epidemiologist

I confirm that I have read this submission and believe that I have an appropriate level of expertise to confirm that it is of an acceptable scientific standard.

## References

[1] : WHO guideline on control and elimination of human schistosomiasis. *World Health Organization* .2022; Reference source 35235279

# Response

**Published:** 2023-04-05

**Authors:** Amy Sturt

**Affiliations:** Veterans Affairs Medical Center Palo Alto, USA

The design was good and adequate but there is a need to improve on the details of some of the aspects of the methods to allow replication by others.  

1. Urine sample was collected during home visit – the authors should provide specific time of urine collection, as this is important in the diagnosis of schistosomiasis. Was the time of collection the same for all participants?

 
*Thank you for the opportunity to clarify. Since visits were performed in the participant’s home, there was not a restriction on the timing of urine sample self-collection and 69.5% (419/603) were performed between 9:00 and 14:00. This has been added from lines 140-142.*  

1. Up to 50mL of urine was centrifuged and examined – the authors should provide the specific amount of urine centrifuged and why 50mL and not 10mL as recommended by WHO (2022), and for ease of comparison with other studies.

 
*Thank you for this question. We have noted a typo in the manuscript, up to 60mL of urine was centrifuged. This has been corrected at line 191. The full details of the urine collection are reported in a previous manuscript. We have referenced this manuscript at line 184 and full details can be found at* *https://journals.plos.org/plosntds/article?id=10.1371/journal.pntd.0008337*
  

1. Figure 1. Flowsheet of cervicovaginal image review – the authors should explain what “images inaccessible” means, particularly as it was among those who attended clinic.

 
*We apologize for any confusion. Due to technical difficulties, Reviewer 1 was not able to open 10 images. In the flowsheet, “images inaccessible” has been changed to “technical difficulty opening images”.*  

1. The authors use the phrase ‘water use’ and ‘water contact’ interchangeably. I think water contact is best for this study.

 
*Thank you for this input and all instances of “water use” have been changed to “water contact”.*

# Peer review: Reviewer response for version 1

**DOI:** 10.21956/wellcomeopenres.20776.r54087

**Published:** 2023-01-27

**Authors:** Louise Arenholt

**Affiliations:** 1 Department of Clinical Medicine, Aalborg University, Aalborg, Denmark; 2 Department of Obstetrics and Gynecology and Centre for Clinical Research, North Denmark Regional Hospital, Hjørring, Denmark

Thank you for the opportunity to review this thorough and well written paper.

 The paper aims to evaluate the agreement on the diagnosis of visual FGS lesions between two expert reviewers. Furthermore, the association between visual presentation of FGS and symptoms of FGS/PCR detection of FGS were evaluated.

 The topic is highly relevant and only a few studies have evaluated the accuracy of visual diagnosis of FGS – even though the visual inspection is the golden standard as also highlighted by the authors.

**Introduction:**

 No comments.

**Methods:**

 I would recommend more detailed information on the hand-held colposcopy and image review. There is no information on how the image was captured and if they had a standard/protocol on which areas to include in the image portfolio (was it only the cervix or also the vaginal walls?). Did they have any criteria for image quality (for example the lighting or zoom level) when capturing the image? How many images were captured per woman? It seems like the reviewers have excluded images on slightly different basis – did you before the review guide the reviewers on how to include or exclude the images? For example, did the reviewers review the images on a computer and were the computers similar (for example resolution on the screen, color setting, brand etc.)? Did they have the opportunity to zoom the image or change color/brightness/contrast/saturation? My experience is that an image can look completely different if you evaluate it on two different computers. A small thing as day light or artificial light can change the image. Could it be different settings/equipment that account for some of the difference between reviewers?

 In the methods section I can’t find information on how women reported the abdominal-genitourinary, and reproductive manifestations (table 4) – questionnaire, interview etc.?

**Results:**

 I am not surprised that you do not find an association between visual-FGS and PCR-FGS. I believe that cevicovaginal lesions are results of chronic inflammation and therefore other tests should be used if an association should be found.

**Discussion:**

 You discuss the study from Madagascar, where gridded image technique was used. And you are correct that all included women in that study were to have FGS lesions. I just need to address two things 1) the presence of rubbery papules was not an inclusion criterion for the study. Therefore, the uncertainty about whether a lesion was present or not was indeed an issue. Some of the reviewers in that study found, that more than 20% of the women had no rubbery papules. 2) the images used for the study were not from the inclusion visit, but from later visits where treatment with Praziquantel had been initiated. For that reason, some of the images could be without FGS lesions.

 I agree with you that consensus on how to evaluate the images is very important. I believe that if consensus had been reached before the reviewing procedure, your results would have been very much different.

Is the work clearly and accurately presented and does it cite the current literature?

Yes

If applicable, is the statistical analysis and its interpretation appropriate?

Yes

Are all the source data underlying the results available to ensure full reproducibility?

Partly

Is the study design appropriate and is the work technically sound?

Yes

Are the conclusions drawn adequately supported by the results?

Yes

Are sufficient details of methods and analysis provided to allow replication by others?

Partly

Reviewer Expertise:

Gynecology and obstetrics. Urogynecology.

I confirm that I have read this submission and believe that I have an appropriate level of expertise to confirm that it is of an acceptable scientific standard, however I have significant reservations, as outlined above.

# Response

**Published:** 2023-04-05

**Authors:** Amy Sturt

**Affiliations:** Veterans Affairs Medical Center Palo Alto, USA

"I would recommend more detailed information on the hand-held colposcopy and image review. There is no information on how the image was captured and if they had a standard/protocol on which areas to include in the image portfolio (was it only the cervix or also the vaginal walls?). Did they have any criteria for image quality (for example the lighting or zoom level) when capturing the image? How many images were captured per woman?"

*Thank you for the opportunity to clarify the colposcopy methods. Images were captured according to a standard protocol and the cervix, anterior fornix, posterior fornix, right lateral cervix, left lateral cervix and adjacent cervical trunk were inspected routinely. The clinician was instructed to take a photograph of each location as well as the cervix. The clinician then examined the anterior and posterior vaginal walls. Per the protocol, the clinician was instructed to capture a minimum of 5 images per participant. All participants were examined in the same clinic room with the overhead lights on. The midwives were allowed to choose to use the in-device lighting or zoom level on the MobileODT colposcope. These details have been added to the manuscript at lines 149-153.*  

 "It seems like the reviewers have excluded images on slightly different basis – did you before the review guide the reviewers on how to include or exclude the images? For example, did the reviewers review the images on a computer and were the computers similar (for example resolution on the screen, color setting, brand etc.)? Did they have the opportunity to zoom the image or change color/brightness/contrast/saturation? My experience is that an image can look completely different if you evaluate it on two different computers. A small thing as day light or artificial light can change the image. Could it be different settings/equipment that account for some of the difference between reviewers?"

*At their discretion, expert reviewers could exclude images that they felt could not be evaluated due to technical issues, image quality, or limited cervical visualization.* *The reviewers independently evaluated the images on desktop computers. The reviewers were able to zoom and make adjustments to the digital images as needed. We feel it is important to consider that in real-world conditions, if a number of technical factors are required to ensure successful image review, the successful roll-out and scale up of this method of diagnosis will be constrained. To replicate real-world conditions, standardized equipment was not provided to the reviewers by the BILHIV study. However, you are correct that we cannot exclude that differences in settings and equipment may theoretically contribute for some differences between reviewers. We have added this as a limitation in the discussion of the manuscript at lines 392 -395.*

 "In the methods section I can’t find information on how women reported the abdominal-genitourinary, and reproductive manifestations (table 4) – questionnaire, interview etc.?" *Women reported symptoms in a structured questionnaire with a community health worker, this is described in line 139.*  

**"Results:**

 I am not surprised that you do not find an association between visual-FGS and PCR-FGS. I believe that cevicovaginal lesions are results of chronic inflammation and therefore other tests should be used if an association should be found."

*We agree that visual-FGS represents a different phenotype than PCR-FGS. Thank you for your input on the results.*

**"Discussion:**

 You discuss the study from Madagascar, where gridded image technique was used. And you are correct that all included women in that study were to have FGS lesions. I just need to address two things 1) the presence of rubbery papules was not an inclusion criterion for the study. Therefore, the uncertainty about whether a lesion was present or not was indeed an issue. Some of the reviewers in that study found, that more than 20% of the women had no rubbery papules. 2) the images used for the study were not from the inclusion visit, but from later visits where treatment with Praziquantel had been initiated. For that reason, some of the images could be without FGS lesions."

*Thank you for the helpful clarifications regarding your outstanding work from Madagascar. In light of the further information you have provided, we have removed the sentences in question in the discussion. These formerly read “However, it is notable that in the Madagascan study, all images were thought to contain FGS lesions, removing the burden of uncertainty and highlighting that in settings where images are known to contain FGS lesions, agreement between reviewers was at best “moderate”.* I agree with you that consensus on how to evaluate the images is very important. I believe that if consensus had been reached before the reviewing procedure, your results would have been very much different. *Thank you for this input and we included the absence of an initial consensus review as a limitation in the discussion at line 407.*
